# Genotypic spectrum and phenotype correlations of *EYS*-associated disease in a Chinese cohort

**DOI:** 10.1038/s41433-021-01794-6

**Published:** 2021-10-23

**Authors:** Feng-Juan Gao, Dan-Dan Wang, Fang-Yuan Hu, Ping Xu, Qing Chang, Jian-Kang Li, Wei Liu, Sheng-Hai Zhang, Ge-Zhi Xu, Ji-Hong Wu

**Affiliations:** 1 Eye Institute, Eye and ENT Hospital, College of Medicine, Fudan University, Shanghai, China; 2 Shanghai Key Laboratory of Visual Impairment and Restoration, Science and Technology Commission of Shanghai Municipality, Shanghai, China; 3 Key Laboratory of Myopia (Fudan University), Chinese Academy of Medical Sciences, National Health Commission, Shanghai, China; 4 BGI-Shenzhen, Shenzhen, Guangdong China; 5 Department of Computer Science, City University of Hong Kong, Kowloon, Hong Kong

**Keywords:** Eye diseases, Hereditary eye disease

## Abstract

**Background:**

To date, certain efforts have been made to investigate the clinical and genetic characteristics of patients with *EYS* mutations. However, data for Chinese patients are limited.

**Objectives:**

To perform a detailed phenotyping and genetic characterization of 55 Chinese patients with *EYS*-RD, and to identify risk factors for these clinical data.

**Methods:**

A total of 55 patients with *EYS*-RD were recruited. Best-corrected visual acuity (BCVA), patient age, age at symptom onset, disease duration, and genetic information were collected.

**Results:**

Thirty-six novel variants, three hot mutations of *EYS* (30.3%, c.6416G>A, c.6557G>A, c.7492G>C) and one hot region (49.06%, Laminin G domains) were identified. In all, 36.84% of the mutations occurred at base G site, and majority of mutations (56.56%) were missense. Late-truncating mutations are significantly more prevalent (41.30%). The mean age of onset was 15.65 ± 14.67 years old; it had no significant correlation with genotype. The average BCVA was 0.73 ± 0.93 LogMAR, and 61.8% of eyes had a BCVA better than 0.52 logMAR. BCVA was positively correlated with disease duration time. The mean MD was 23.18 ± 7.34 dB, MD showed a significant correlation with genotype and age. Cataract was present in 56.45% of patients, and 42.59% of patients showed an absence of pigmentation in the retina. Cataract and hyperpigmentation both showed a significant correlation with age.

**Conclusions:**

*EYS-RD* is associated with a moderate phenotype with onset around adolescence, but great variability. Our study largely enhances the current knowledge of phenotypic and genotypic characteristics of *EYS*-RD, which could pave the way for better management of these patients.

## Introduction

Eyes shut homolog (*EYS*; OMIM: 612424), spanning approximately 2 Mb of chr6q12 and consisting of 44 exons, was first reported in 2008 as a disease-causing gene for autosomal recessive retinitis pigmentosa (RP). *EYS* is the largest gene in the eye and encodes a protein with 3156 amino acids that is predominantly expressed in the retina and plays an essential role in the morphogenesis of photoreceptors [1–4]. The EYS protein contains 27 epidermal growth factor-like (EGF) domains and five laminin G-like domains, which are highly conserved [1].

Mutations in *EYS* are associated with several phenotypes, such as RP, cone–rod dystrophy (CRD), and Leber congenital amaurosis (LCA), and we collectively call these phenotypes *EYS-*associated retinal disease (*EYS*-RD) here [5–8]. Our prior study reported that the prevalence of *EYS* mutations in patients with inherited retinal dystrophies (IRDs) was 7% in the Chinese population, and *EYS* mutations were ranked as the third most common genetic mutation in patients with IRD [7]. The high prevalence of *EYS*-RD makes further in-depth research on its clinical and genetic characteristics more urgent and significant.

To date, certain efforts have been made to investigate the clinical and genetic characteristics of patients with *EYS* mutations. However, most studies were conducted in Japanese cohorts [5, 9–11], and data for Chinese patients are limited [12]. In this study, 55 patients with *EYS*-RD were enrolled, which is the largest cohort to our knowledge. The purpose of this study was to perform a detailed phenotyping and genetic characterization of 55 Chinese patients with *EYS-RD*, and to identify risk factors for these conditions.

## Materials and methods

### Subjects and ethical statement

The protocol of this study adhered to the tenets of the Declaration of Helsinki and was approved by the Ethics Committee of the Eye and Ear, Nose and Throat (ENT) Hospital of Fudan University. A total of 55 patients from 46 families with *EYS*-RD were enrolled from the Eye and ENT Hospital of Fudan University between January 2017 and December 2019; all of the participants were of Chinese descent. Of the patients, 38 were mentioned in our previous report [7]. Written informed consent was obtained from all subjects.

### Genetic analysis

Genomic DNA was extracted from the peripheral blood of all affected subjects and the available family members. Molecular testing and data analysis were performed as previously reported [7, 13–16]. We designed a high-throughput targeted enrichment approach to exon-capture regions of 762 genes involved in common inherited eye diseases. The capture panel was custom designed and produced by the Beijing Genomics Institute (BGI, Shenzhen, China) (Supplementary Table 1). For acquisition of probe sequences, we obtained the exon sequence and its flank ±30 bp of 762 genes from a reference human genome (UCSC hg 38). Each reference sequence begins at one end of a reference sequence and selects the reference sequence of the predetermined length to obtain the probe sequence so that the last total probe can cover the reference sequence at least once. The probe length of the panel is 90 nt; the total target area obtained is 2.3 M. On average, the mean coverage depth was more than 400×, and the coverage of target region was ~99.9% using BGISEQ-2000. Subsequent points for sample quality control were also added to the probe design process. The exon deletion was found by CapCNV analysis followed by CNVkit protocol (https://cnvkit.readthedocs.io/en/stable/pipeline.html). The potential pathogenicity of the variants was interpreted according to the American College of Medical Genetics.

Patients were divided into three groups depending on the number and type of the identified variants, as previously described [17–19]. The genotype A group (severe) included patients with ≥2 null variants, the genotype B group included patients with 1 null variant and 1 variant that was a missense or in-frame insertion/deletion, and the genotype C group (moderate) included patients with no null variant but ≥2 variants that were missense or in-frame insertions/deletions. Null variants were those that would be predicted to affect splicing or to introduce a premature truncating codon in the protein, such as nonsense, frameshift, exonic, or intronic variants with significant splice-site alteration [19, 20].

### Clinical examination

Clinical information, including medical history, family history, ethnicity, chief complaints of visual symptoms, onset of disease, best Snellen-corrected visual acuity (BCVA, converted to equivalent value of logarithm of minimal angle of resolution (logMAR) unit), slit-lamp biomicroscopy of anterior and posterior segments, wide-field fundus imaging (Optos PLC, Dunfermline, United Kingdom), fundus autofluorescence (FAF, Spectralis HRA COCT; Heidelberg, Germany), visual field (VF, Humphrey Visual Field Analyzer, Carl Zeiss Inc., Dublin, CA, USA), swept-domain optical coherence tomography (Spectralis HRA+OCT, Heidelberg Engineering Inc., Heidelberg, Germany), and full-field electroretinography (according to the standards of the International Society for Clinical Electrophysiology of Vision; www.iscev.org) were assessed. VF was assessed by 30-2 Swedish Interactive Threshold Algorithm (SITA) Fast Programs to measure 30° temporally and nasally and test 76 points. The VF data were excluded if fixation loss and false-positive and false-negative response rates were greater than 20%. The average depression of visual sensitivity was estimated by the mean deviation (MD). To provide numeric values for low BCVAs, the following conversions were performed: light perception, 4 logMAR; hand movements, 3 logMAR; and counting fingers, 2 logMAR.

### Statistical analysis

The statistical analysis was carried out using SPSS software version 22.0 (IBM Corp., Armonk, NY). Statistical significance for all statistical tests was set at *P* < 0.05. Measurement values of the groups were compared using the *t*-test and one-way ANOVA. Correlations between pairs of measurements were evaluated using the Pearson or Spearman test, as appropriate. Linear mixed models were used to assess the impact of clinical data (age, age of symptom onset, disease duration time, BCVA, MD, retinal hyperpigmentation, and cataract) and genetic data on certain data.

## Results

### Cohort characteristics

In total, 55 affected subjects (females 21, males 34) from 46 families with *EYS*-RD were enrolled; all of the participants were of Chinese descent. Pedigrees and mutations of the 46 families are available in Supplementary Fig. 1. The demographic and clinical characteristics are summarized in Table 1. Of the 55 patients, 53 were diagnosed with RP and 2 had LCA. The median age of these subjects was 38.0 years old at the examination (mean, 39.96 ± 14.87; range, 8.0–74.0), and the mean disease duration was 24.13 ± 15.40 years (median 22.5; range, 2.0–66.0).

**Table 1:** Baseline demographic and clinical characteristics of all participants.

Characteristics	Data
No. patients (%)	
Total	55
Female/male	21/34 (38.2%/61.8%)
Mean age (yr)	39.96 ± 14.87
Genotype A	11 (20.0%)
Genotype B	30 (54.5%)
Genotype C	14 (25.5%)
Onset ≤5 yr^a^	14 (32.6%)
Onset 6–18 yr	16 (37.2%)
Onset 19–49 yr	12 (27.9%)
Onset ≥50 yr	1 (2.3%)
No. eyes (%)^b^	76
HM-1.3 (BCVA)	13 (17.1%)
1.3–1 (BCVA)	3 (3.9%)
1–0.52 (BCVA)	13 (17.1%)
0.52–0.3 (BCVA)	19 (25.0%)
0.3–0 (BCVA)	28 (36.8%)
Age of onset (yr)	
Average	15.65 ± 14.27
Genotype A	13.44 ± 5.58
Genotype B	18.87 ± 16.61
Genotype C	8.88 ± 11.12
BCVA logMAR	
Average	0.73 ± 0.93
Age ≤ 18 yr	1.14 ± 1.44
Age 19–49 yr	0.30 ± 0.24
Age >50 yr	1.57 ± 1.12
Genotype A	0.42 ± 0.27
Genotype B	0.80 ± 0.93
Genotype C	0.87 ± 1.27
Mean disease duration (yr)	24.13 ± 15.40
Cataract (eyes)^c^	35 (56.45%)
Mean age (yr)	43.76 ± 16.46
Mean BCVA logMAR	1.02 ± 1.07
Mean disease duration (yr)	27.67 ± 16.52
Genotype A (eyes)	12
Genotype B (eyes)	34
Genotype C (eyes)	16
Retinal hyperpigmentation (eyes)	62 (57.41%)
Mean age (yr)	41.11 ± 15.59
Mean BCVA logMAR	0.48 ± 0.28
Mean disease duration (yr)	25.25 ± 13.67
Genotype A (eyes)	14
Genotype B (eyes)	32
Genotype C (eyes)	16

*No*. number, *BCVA,* best-corrected visual acuity, *HM* hand moving, *yr* years.

^a^Age at symptom onset was available in 43 patients (86 eyes). ^b^BCVA was available in 38 patients (76 eyes). ^c^Condition of the lens was available in 31 patients (62 eyes). Pigmentary changes were available in all patients (108 eyes).

### Genetic analyses

**Fig. 1:**
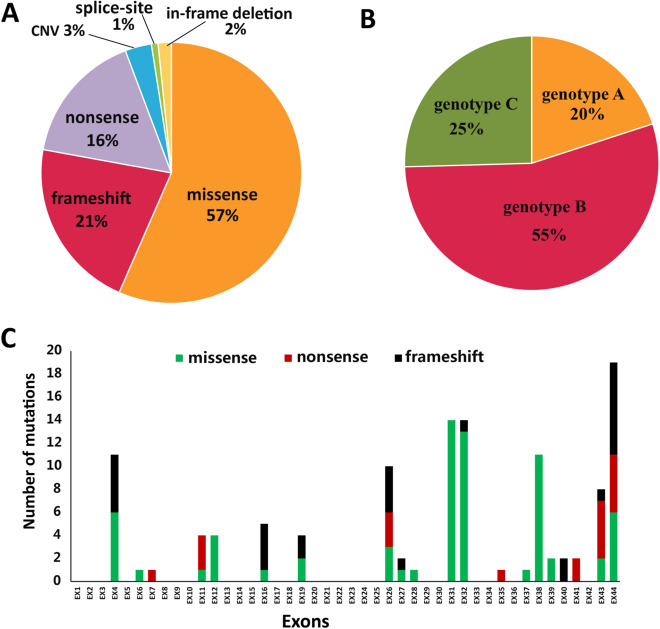
Overview of the pathological EYS mutations identified in this study. **A** The proportion of different mutation types in EYS-associated retinal disease (EYS-RD). A total of 56.56% were missense, 16.39% were nonsense, 21.31% were frameshift, 1.64% were in-frame deletions, 3.28% were copy-number variations (CNVs), and 0.82% were splice-site mutations. **B** The proportion of different genotypes in EYS-RD patients. **C** Distribution of the EYS variants (missense, nonsense, and frameshift) on different exons. Late-truncating pathogenic variants (exons 43–44) are significantly more prevalent (41.30%).

**Fig. 2:**
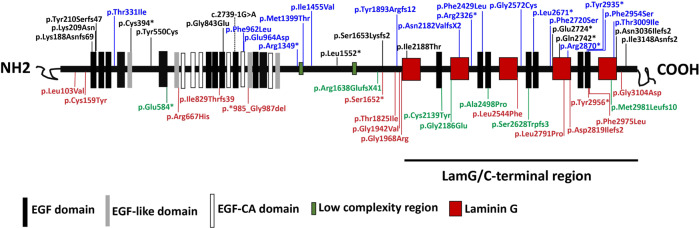
Schematic representation of EYS protein domains and the distribution of variants identified in this study. Variants identified in patients with and without cataracts are listed in red (*n* = 15) and black (*n* = 14), respectively. Variants identified both in patients with and without cataracts are listed in green (*n* = 7). Variants identified in patients with unknown conditions of the lens are listed in blue (*n* = 17). The majority of variants (49.06%) harbored by the cohort were spatially distributed across the laminin G domains toward the carboxy (−COOH) end of the protein. Color figure online.

Cosegregation analysis was performed in 46 families. Pedigrees showing the clinical and genetic status of the 46 families are available in Supplementary Fig. 1. A total of 122 alleles representing 56 distinct variants were identified, and of these alleles, 36 were novel (Supplementary Table 2). Sanger verification was performed on all mutations, and Supplementary Fig. 2 shows the partial results of Sanger sequencing. Of the 122 variants identified in these patients, the majority of pathogenic defects (58.20%, *n* = 71) were missense variants (56.56%, *n* = 69) or in-frame deletions (1.64%, *n* = 2), and 41.80% (*n* = 51) were nonsense (16.39%, *n* = 20), frameshift (21.31%, *n* = 26), splice-site (0.82%, *n* = 1) mutations or copy-number variations (CNVs, 3.28%, *n* = 4) that severely affected protein function (Fig. 1A). Of the 46 truncating mutations, 13 (28.26%) were located in exon 44 and 6 (13.04%) were located in exon 43 (Fig. 1C), indicating that late-truncating pathogenic variants (exons 43–44) are significantly more prevalent (41.30%). Eleven patients (20.0%) were in the genotype A group, 30 (54.5%) were in the genotype B group, and 14 (25.5%) were in the genotype C group (Fig. 1B). The most prevalent alleles were c.6416G>A p.Cys2139Tyr (11.5%, *n* = 14), c.6557G>A p.Gly2186Glu (9.8%, *n* = 12), and c.7492G>C p.Ala2498Pro (9.0%, *n* = 11), accounting for 30.3% of all alleles (37/122), suggesting that they are hotspot mutations of *EYS* in the Chinese population. In addition, 15 variants were found in patients presenting with cataracts, 7 were found both in patients with and without cataracts, and 14 were found in patients without cataracts at the time of examinations. Most variants (49.06%) harbored by the cohort were spatially distributed across the laminin G domains toward the carboxy (−COOH) end of the protein (Fig. 2). One striking feature of the point mutations (*n* = 38) is that 36.84% (*n* = 14) of them occurred at the base G site.

### Phenotypic characterization and associated risk factors

#### Age of symptom onset

**Fig. 3:**
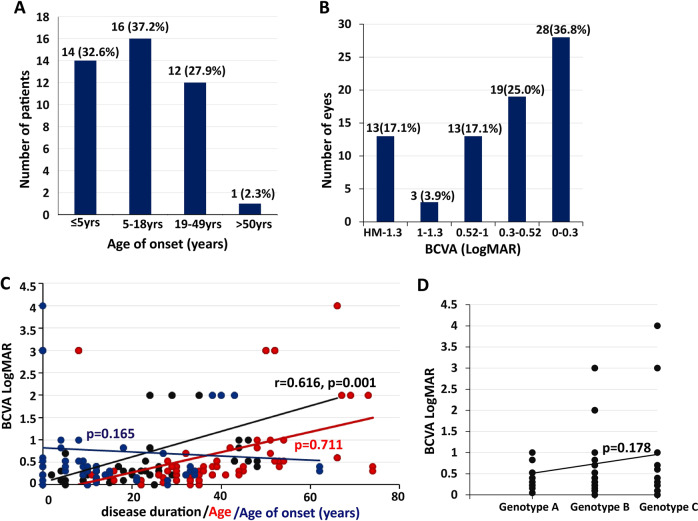
Phenotypic characterization and associated risk factors. **A** Number of patients in different age of onset groups. **B** Number of eyes in different BCVA logMAR groups. **C** Correlation of BCVA logMAR with age, age of onset, and disease duration. LogMAR BCVA was positively correlated with disease duration (coefficient: 0.616, *P* = 0.001) but had no significant associations with age (*P* = 0.711) or age of symptom onset (*P* = 0.165). Disease duration is marked in black (font, line, or circle), age is marked in red, and age of symptom onset is marked in blue. **D** Correlations between BCVA logMAR values and genotype (*P* = 0.178). BCVA best-corrected visual acuity. Color figure online.

Age of symptom onset was available for 43 patients (86 eyes). Disease onset presented a wide spectrum from birth to 62 years of age (mean 15.65 ± 14.67, median 12). In detail, 32.6% (*n* = 14) of patients had an onset earlier than 5 years of age (infant onset), 37.2% (*n* = 16) of patients had an onset between 6 and 18 years of age (juvenile onset), 27.9% (*n* = 12) of patients had an onset between 19 and 49 years of age, and only 1 (2.3%) patient had an onset at age 62 years (Fig. 3A). Spearman analysis revealed that age of onset had no significant correlation with genotype (coefficient: −0.163, *P* = 0.149).

#### BCVA

BCVA data were available for 38 patients (76 eyes). Measured BCVAs ranged from 0 logMAR to light perception (mean 0.73 ± 0.93), and 17.1% (13/76) of eyes had a BCVA worse than logMAR 1.3, 21.1% (16/76) of eyes had a BCVA worse than 1 logMAR, and 61.8% (47/76) of eyes had a BCVA better than 0.52 logMAR (Fig. 3B and Table 1). To further investigate the impact of clinical data (age, disease duration, age of symptom onset, VF, retinal hyperpigmentation, and genotype) on BCVA, a linear mixed-effects model was used. The results showed that logMAR BCVA was positively correlated with disease duration (coefficient: 0.616, *P* = 0.001) but had no significant associations with age (*P* = 0.711), age of symptom onset (*P* = 0.165), MD (*P* = 0.065), retinal hyperpigmentation (*P* = 0.423), or genotype (*P* = 0.178) (Fig. 3C, D).

#### Visual field

**Fig. 4:**
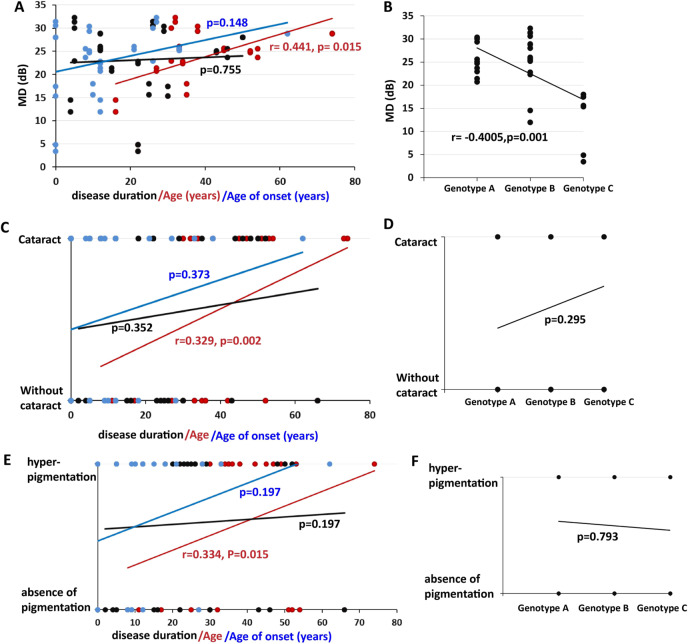
Correlation of MD, hyperpigmentation, cataract, age, age of onset, disease duration and genotype. **A** Correlation of MD with age, age of onset, and disease duration. MD showed a significant correlation with age (coefficient: 0.441, *P* = 0.015) but was not correlated with disease duration (*P* = 0.755) or age of symptom onset (*P* = 0.148). **B** Correlations between MD and genotype (*P* = 0.178). MD showed a significant correlation with genotype (coefficient: −0.4005, *P* = 0.001). **C** Correlation of cataracts with age, age of onset, and disease duration. Cataracts showed a significant correlation with age (coefficient: 0.329, *P* = 0.002), but there was no significant association of cataracts with age of onset (*P* = 0.373) or disease duration (*P* = 0.352). **D** Correlations between cataract and genotype (*P* = 0.295). **E** Correlation of hyperpigmentation with age, age of onset, and disease duration. Hyperpigmentation showed a significant correlation with age (coefficient: 0.334, *P* = 0.015), but there was no significant association of hyperpigmentation with age of onset (*P* = 0.197) or disease duration (*P* = 0.197). **F** Correlations between cataract and genotype (*P* = 0.793). Disease duration is marked in black (font, line, or circle), age is marked in red, and age of symptom onset is marked in blue. MD mean deviation. Color figure online.

Severe cataracts could affect the VF test results, so patients with severe cataracts were excluded from the VF analysis. Therefore, VF was available for only 16 patients (32 eyes). The mean MD was 23.18 ± 7.34 dB (range 3.44–32.31, median 25.15). Linear regression analysis showed that MD was not correlated with disease duration (*P* = 0.755), age of symptom onset (*P* = 0.148), or BCVA (*P* = 0.075) but showed a significant correlation with genotype (coefficient: −0.4005, *P* = 0.001) and age (coefficient: 0.441, *P* = 0.015) (Fig. 4A, B). Severe genotype and older age were associated with severe VF defects.

#### Cataract

The condition of the lens was available in 31 patients (62 eyes). Cataracts are a common finding in this cohort of patients and were found in all age groups (range 8–74 years old, mean 43.76 ± 16.46 years old). Of the 62 eyes, 35 (56.45%) showed varying degrees of cataracts, with a mean BCVA of 0.48 ± 0.28 logMAR, and the median disease duration was 25.25 ± 13.67 years (Table 1). Patients without cataracts (31.71 ± 14.23 years old) were younger than patients who had cataracts (*P* = 0.003), and BCVA was worse in patients who had cataracts (1.02 ± 1.07 vs 0.46 ± 0.79 logMAR, *P* = 0.027). However, age of onset (*P* = 0.096) and disease duration (*P* = 0.352) were not significantly different between the two groups. There were no significant associations of cataracts with genotype (*P* = 0.295), age of onset (*P* = 0.373) or disease duration (*P* = 0.352), but cataracts showed a significant correlation with age (coefficient: 0.329, *P* = 0.002) and BCVA (coefficient: 0.348, *P* = 0.009) (Fig. 4C, D).

### Retinal hyperpigmentation

Data on pigment change in the retina were available for all patients (108 eyes). Of the 108 eyes, 46 (42.59%) showed an absence of pigmentation in the retina, while 62 eyes showed hyperpigmentation (Table 1 and Supplementary Fig. 3). The mean age of the patients who showed hyperpigmentation was 41.11 ± 15.59 years old, the mean BCVA was 0.48 ± 0.28 logMAR, and the median disease duration was 25.25 ± 13.67 years. Patients without pigmentation (31.62 ± 18.06 years old) were younger than those with hyperpigmentation (*P* = 0.024), and the age of symptom onset was earlier in patients without hyperpigmentation (7.82 ± 7.88 vs 15.00 ± 15.50 years old), but BCVA (*P* = 0.524) and disease duration (*P* = 0.705) were not significantly different between the two groups. Correlation analysis revealed that there were no significant associations of hyperpigmentation with genotype (*P* = 0.793), disease duration (*P* = 0.197), BCVA (*P* = 0.446), or MD (*P* = 0.056), but hyperpigmentation showed a significant correlation with age (coefficient: 0.334, *P* = 0.015) (Fig. 4E, F). These results indicate that older age was associated with pigmentary changes.

## Discussion

*EYS*-RD is one of the most prevalent IRDs in Japanese and European populations [10, 21–25]. The proportion has been reported to be 5% in the Netherlands and Canada, 7% in Israel, 11% in the UK, 12% in France, 17% in Spain, and 18–23.5% in Japanese cohorts of RP patients. The vast majority of mutations were truncating, and the most prevalent variants were c.4957_4958insA, c.2528G>A, and c.8868C>A in the Japanese population [5, 10, 26]. However, we found that the prevalence of *EYS* mutations in Chinese patients was 7% in our previous study, ranked as the third most common gene detected in this cohort of patients with IRD (*n* = 896) [7]; the majority of pathogenic defects (56.56%) were missense variants, and the most prevalent variants were c.6416G>A (11.5%), c.6557G>A (9.8%), and c.7492G>C (9.0%). Only one allele of c.4957_4958insA, c.2528G>A, and c.8868C>A was found in our cohort. These results indicate a different founder mutation of *EYS* in our population. Of special interest, we found for the first time that late-truncating pathogenic variants are significantly more prevalent (41.30%) in the *EYS* gene, and 36.84% of point mutations occurred at the base G site. The interpretation of these findings is challenging, which might suggest a site-specific effect, or this variability may be related to underlying genetic variants. Further investigation of these mechanisms should promote better understanding.

Of the 55 patients, 53 (96.36%) were diagnosed with RP, 2 (3.64%) had LCA, and no CRD phenotype was found. We assumed that *EYS*-associated CRD was rare in the Chinese population. In a recent study of a Japanese cohort, the median age of symptom onset was 19 years (range, 14–62 years) [5], while in a Canadian cohort, the median age of symptom onset was 21 years (range, 1–65 years) [23]. In our cohort, the median age of symptom onset was younger than in the previous two reports (mean 15.65 ± 14.67, median 12, range 0–62 years). Consistent with previous studies, age of symptom onset had no significant correlation with genotype [5, 23], suggesting the involvement of other modifying genes. The mean BCVA was 0.73 ± 0.93, and 61.8% of eyes had a BCVA better than 0.52 logMAR. These results would place *EYS*-RD in the milder spectrum of disease. To date, limited data have documented factors affecting BCVA. Our study showed that longer duration was associated with poorer BCVA, but BCVA showed no significant associations with age, age of symptom onset, MD, retinal hyperpigmentation, or genotype. Unlike BCVA, MD showed a significant correlation with genotype and age but not with disease duration or age of symptom onset. One possible explanation is that aqueous flare is increased and retinal degeneration progresses in older age, especially in RP patients [27–29]. Aqueous flares were negatively correlated with the residual VF area, but no correlation was detected with BCVA [28, 29]. For this reason, severe genotype and older age were associated with severe VF defects but not with BCVA. Although our study did not investigate aqueous flares, understanding the predictive factors associated with visual acuity (VA) and VF may help ophthalmologists or geneticists manage patients’ expectations and provide more accurate genetic counseling. Moreover, these data indicate that gene, stem cell, or drug therapy should be performed as soon as possible in the course of the disease.

Posterior subcapsular cataract occurs in approximately 45% of RP patients [30, 31]. The underlying mechanism is currently unknown, although a possible association with inflammation was proposed [32]. In this study, we found that cataracts were observed in 56.45% of *EYS*-RD patients, and these patients were distributed in all age groups (range 8–74, mean 43.76 ± 16.46 years). This finding is consistent with prior findings in a Japanese cohort, which revealed that cataracts were observed in seven of the ten affected subjects [9]. These results suggest that the occurrence of cataracts in patients with RP may be associated with genetic predisposition. However, there were no significant associations of cataracts with genotype or disease duration, but cataracts showed a significant correlation with age. This can also be explained by the fact that the inflammatory response increased with age, which is a significant risk factor for cataract formation [32]. One of the characteristic fundoscopic features of RP is intraretinal pigment (bone spicule) migration in regions of photoreceptor degeneration. However, the absence or scarcity of typical RP-related hyperpigmentation has also been described in several RP subtypes [33]. The reason for this is unclear, although myopic degeneration may be a factor in some patients [34]. In this analysis, 42.59% of subjects showed an absence of pigmentation, and they were younger than those who showed hyperpigmentation. Correlation analysis revealed that age was a risk factor for hyperpigmentation, and older age was associated with pigment formation. Takahashi et al. [35] reported that as the age of RP patients increased, there was a higher prevalence of pigment migration, reaching over 95% after 40 years of age [35]. Therefore, as age increases, some patients who do not present pigments may develop pigments. However, there is another possibility that no pigment will be observed in their lifetime due to the relatively milder pathogenicity of *EYS*.

We are aware that the present study has several limitations. First, our study participants were enrolled from a single tertiary referral center (Eye and ENT Hospital of Fudan University), and this was a cross-sectional study. Multicentre and longitudinal follow-up studies may be conducted in the near future. Second, due to the rare nature of *EYS*-RD, only 55 individuals were available for inclusion, and this small sample size limits the statistical significance of our results. Additionally, age at symptom onset is a subjective measure, and it is difficult to obtain the exact values.

In conclusion, we explored the clinical and genetic characteristics of EYS-RD in a Chinese cohort. Three hot mutations, 36 novel variants, and two unreported mutational features of EYS were identified. Baseline values and correlations of these clinical data (age, age of onset, disease duration time, BCVA, MD, cataract, and pigment change in the retina) were uncovered. To the best of our knowledge, this is the largest clinical and genetic analysis of EYS-RD in the Chinese population. Our data will serve as a well-founded reference for genetic counseling, better management of these patients, and putative therapeutic approaches.

### Summary table

#### What was known before

- To date, certain efforts have been made to investigate the clinical and genetic characteristics of patients with EYS mutations. However, data for Chinese patients are limited.

#### What this study adds

- Our study largely enhances the current knowledge of phenotypic and genotypic characteristics of EYS-RD in a cohort of Chinese patients, which could pave the way for better management of these patients.

## Supplementary information

Supplementary Figure 1 - Pedigrees of the 45 families.

Supplementary Figure 2 - Sanger sequencing or polymerase chain reaction results of partial EYS variants identified in this study

Supplementary Figure 3 - Representative pictures of patients with or without retinal pigment, including OCT, and VF.

Supplementary Table 1. Details of the 762 genes in the panel

Supplementary Table 2. Genetic and demographic data of patients in this study

## Data Availability

All pathogenic variants identified in this study are provided as supplementary materials; further data that support the findings of this study are available from the corresponding author on reasonable request.
