# Supplementary figures and images for: Genotypic spectrum and phenotype correlations of EYS-associated disease in a Chinese cohort
Source: Eye (Lond). 2021 Oct 23;36(11):2122–9. doi: 10.1038/s41433-021-01794-6 (PMC9581949; doi:10.1038/s41433-021-01794-6)

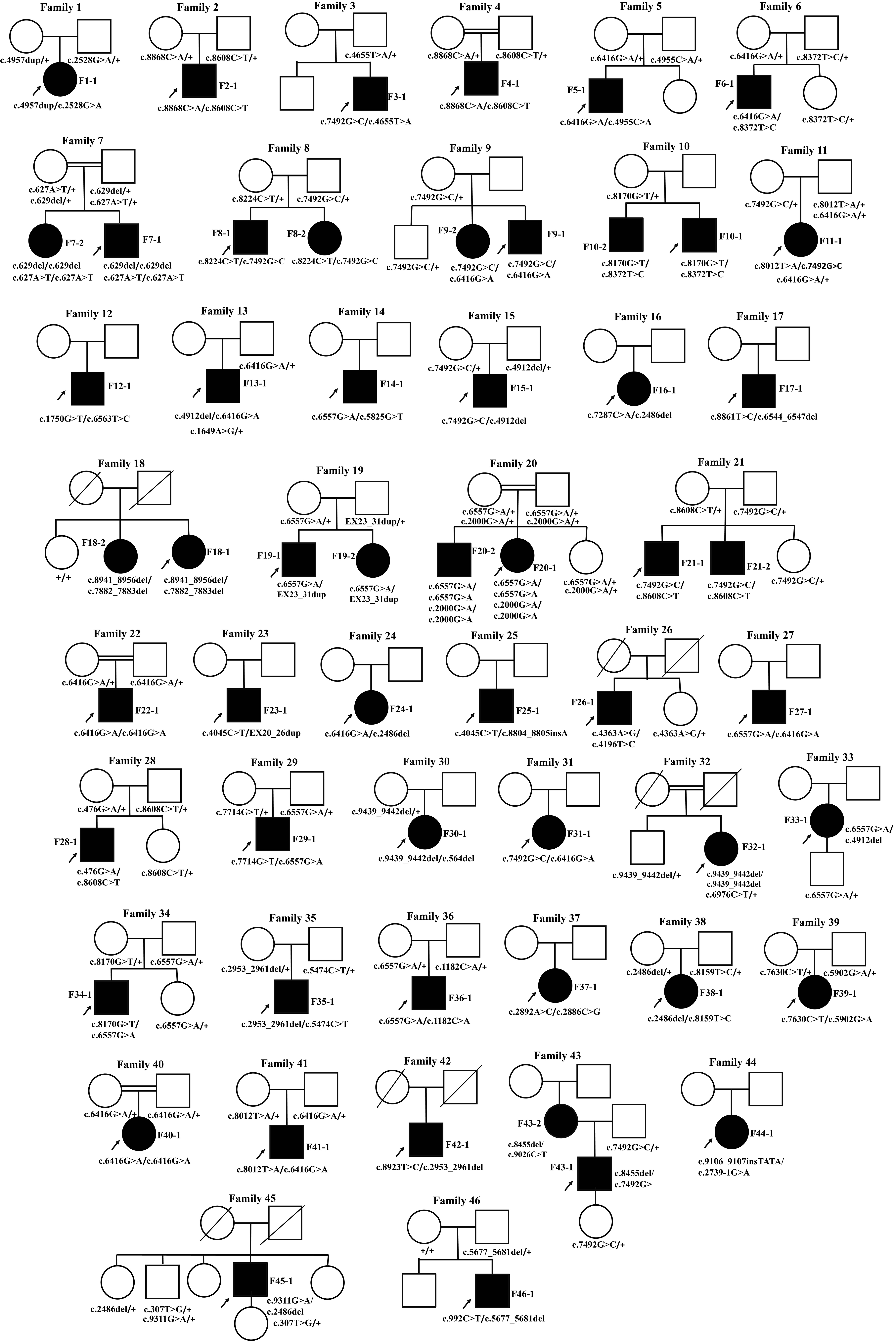

Supplement: Supplementary file 1 — Supplementary Figure 1 - Pedigrees of the 45 families. [file 41433_2021_1794_MOESM1_ESM.jpg]

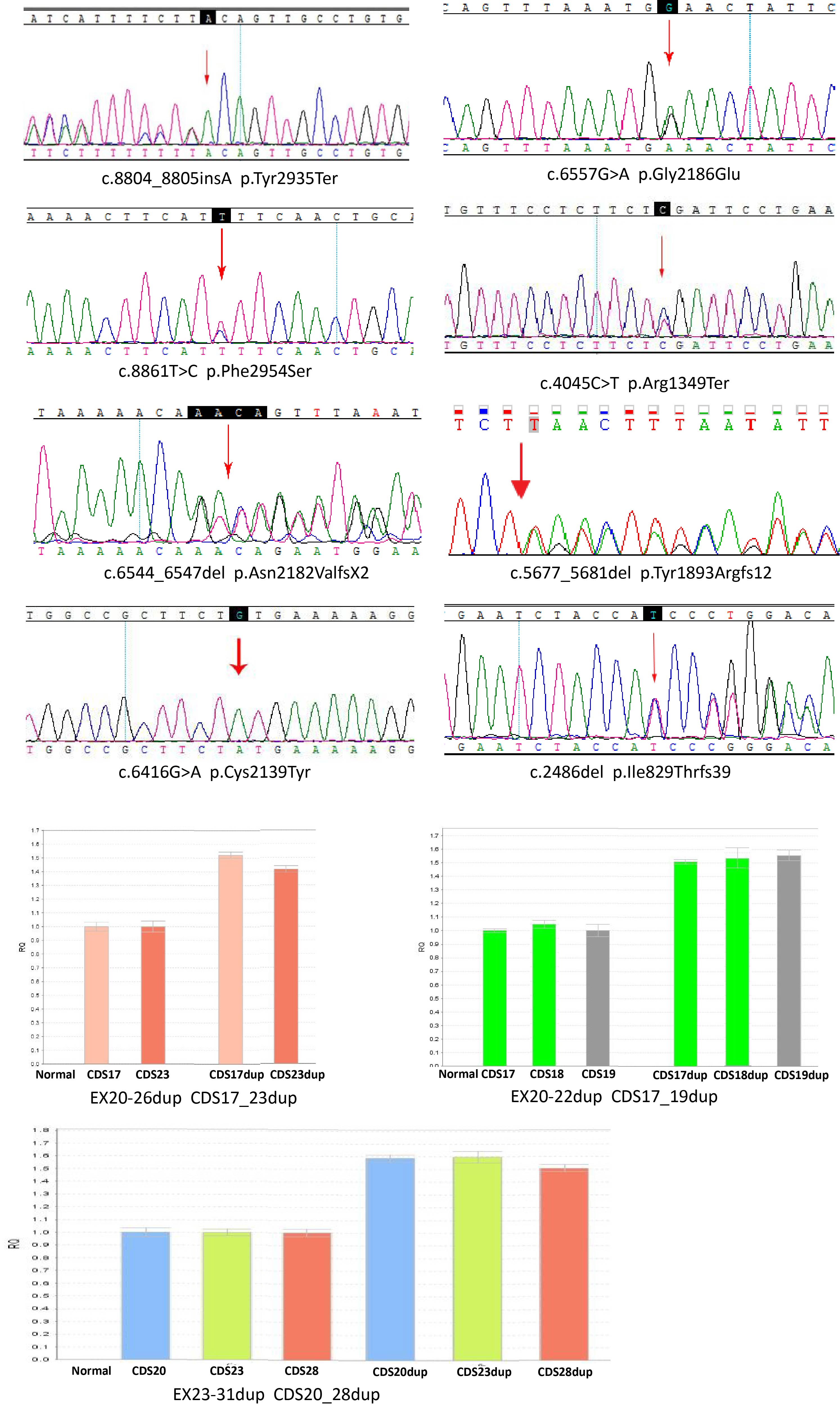

Supplement: Supplementary file 2 — Supplementary Figure 2 - Sanger sequencing or polymerase chain reaction results of partial EYS variants identified in this study [file 41433_2021_1794_MOESM2_ESM.jpg]
