# Supplementary material for: Genotypic spectrum and phenotype correlations of EYS-associated disease in a Chinese cohort
Source: Eye (Lond). 2021 Oct 23;36(11):2122–9. doi: 10.1038/s41433-021-01794-6 (PMC9581949; doi:10.1038/s41433-021-01794-6)
Supplement: Supplementary file 4 — Supplementary Table 1. Details of the 762 genes in the panel [file 41433_2021_1794_MOESM4_ESM.pdf]

Table 1. Details of the 762 genes in the panel.

Gene	OMIM	Subgroup
GJA8	600897	Abnormality of the lens
CRYBA1	123610	Abnormality of the lens
PITX3	602669	Abnormality of the lens
BFSP2	603212	Abnormality of the lens
GCNT2	600429	Abnormality of the lens
GJA3	121015	Abnormality of the lens
MIP	154050	Abnormality of the lens
CRYAB	123590	Abnormality of the lens
CRYBB1	600929	Abnormality of the lens
FYCO1	607182	Abnormality of the lens
LIM2	154045	Abnormality of the lens
CRYGC	123680	Abnormality of the lens
CRYGS	123730	Abnormality of the lens
MAF	177075	Abnormality of the lens
CRYBB3	123630	Abnormality of the lens
CRYBA4	123631	Abnormality of the lens
CRYBB2	123620	Abnormality of the lens
VIM	193060	Abnormality of the lens
CHMP4B	610897	Abnormality of the lens
BFSP1	603307	Abnormality of the lens
TDRD7	611258	Abnormality of the lens
AGK	610345	Abnormality of the lens
CRYGB	123670	Abnormality of the lens
CRYGD	123690	Abnormality of the lens
NHS	300457	Abnormality of the lens
WFS1	606201	Abnormality of the lens
CRYBA2	600836	Abnormality of the lens
HSF4	602438	Abnormality of the lens
EPHA2	176946	Abnormality of the lens
CRYAA	123580	Abnormality of the lens
IARS2	612801	Abnormality of the lens
MSMO1	607545	Abnormality of the lens
CAV1	601047	Abnormality of the lens
SLC33A1	603690	Abnormality of the lens
FOXE3	601094	Abnormality of the lens
ADAMTSL4	610113	Abnormality of the lens
SLC16A12	611910	Abnormality of the lens
FBN1	134797	Abnormality of the lens
ADAMTS10	608990	Abnormality of the lens
ADAMTS17	607511	Abnormality of the lens
DCN	125255	Corneal dystrophy
KERA	603288	Corneal dystrophy
TGFBI	601692	Corneal dystrophy
TACSTD2	137290	Corneal dystrophy
KRT12	601687	Corneal dystrophy
VSX1	605020	Corneal dystrophy
SLC4A11	610206	Corneal dystrophy
PIKFYVE	609414	Corneal dystrophy
PXDN	605158	Corneal dystrophy
COL8A2	120252	Corneal dystrophy
TCF4	602272	Corneal dystrophy
ZEB1	189909	Corneal dystrophy
CHST6	605294	Corneal dystrophy

KRT3	148043	Corneal dystrophy
CHRDL1	300350	Corneal dystrophy
UBIAD1	611632	Corneal dystrophy
ASB10	615054	Glaucoma
MYOC	601652	Glaucoma
OPTN	602432	Glaucoma
WDR36	609669	Glaucoma
NTF4	162662	Glaucoma
CYP1B1	601771	Glaucoma
LTBP2	602091	Glaucoma
ATF6	605537	Inherited retinal dystrophy
CNGA3	600053	Inherited retinal dystrophy
CNGB3	605080	Inherited retinal dystrophy
GNAT2	139340	Inherited retinal dystrophy
PDE6H	601190	Inherited retinal dystrophy
HMCN1	608548	Inherited retinal dystrophy
TLR4	603030	Inherited retinal dystrophy
CST3	604312	Inherited retinal dystrophy
CX3CR1	601470	Inherited retinal dystrophy
CFI	217030	Inherited retinal dystrophy
C2	613927	Inherited retinal dystrophy
CFB	138470	Inherited retinal dystrophy
C9	120940	Inherited retinal dystrophy
FBLN5	604580	Inherited retinal dystrophy
CFH	134370	Inherited retinal dystrophy
ERCC6	609413	Inherited retinal dystrophy
HTRA1	602194	Inherited retinal dystrophy
ARMS2	611313	Inherited retinal dystrophy
C3	120700	Inherited retinal dystrophy
ALMS1	606844	Inherited retinal dystrophy
GABRB1	137190	Inherited retinal dystrophy
IFT27	615870	Inherited retinal dystrophy
BBS1	209901	Inherited retinal dystrophy
BBS10	610148	Inherited retinal dystrophy
TRIM32	602290	Inherited retinal dystrophy
BBS12	610683	Inherited retinal dystrophy
MKS1	609883	Inherited retinal dystrophy
WDPCP	613580	Inherited retinal dystrophy
SDCCAG8	613524	Inherited retinal dystrophy
LZTFL1	606568	Inherited retinal dystrophy
BBIP1	613605	Inherited retinal dystrophy
BBS2	606151	Inherited retinal dystrophy
BBS4	600374	Inherited retinal dystrophy
BBS5	603650	Inherited retinal dystrophy
MKKS	604896	Inherited retinal dystrophy
BBS7	607590	Inherited retinal dystrophy
BBS9	607968	Inherited retinal dystrophy
PLA2G5	601192	Inherited retinal dystrophy
OPN1MW	300822	Inherited retinal dystrophy
RLBP1	180090	Inherited retinal dystrophy
PNPLA6	603197	Inherited retinal dystrophy
KIF11	148760	Inherited retinal dystrophy
CHM	303100	Inherited retinal dystrophy
TMEM67	609884	Inherited retinal dystrophy

OPN1LW	300824	Inherited retinal dystrophy
DRAM2	613360	Inherited retinal dystrophy
PCYT1A	123695	Inherited retinal dystrophy
UNC119	604011	Inherited retinal dystrophy
C21orf2	603191	Inherited retinal dystrophy
RAX2	610362	Inherited retinal dystrophy
GUCA1A	600364	Inherited retinal dystrophy
RAB28	612994	Inherited retinal dystrophy
TTLL5	612268	Inherited retinal dystrophy
POC1B	614784	Inherited retinal dystrophy
PDE6C	600827	Inherited retinal dystrophy
PITPNM3	608921	Inherited retinal dystrophy
RIMS1	606629	Inherited retinal dystrophy
ADAM9	602713	Inherited retinal dystrophy
ACBD5	616618	Inherited retinal dystrophy
GNAT1	139330	Inherited retinal dystrophy
GRK1	180381	Inherited retinal dystrophy
CACNA1F	300110	Inherited retinal dystrophy
NYX	300278	Inherited retinal dystrophy
TRPM1	603576	Inherited retinal dystrophy
SLC24A1	603617	Inherited retinal dystrophy
GRM6	604096	Inherited retinal dystrophy
CABP4	608965	Inherited retinal dystrophy
GPR179	614515	Inherited retinal dystrophy
LRIT3	615004	Inherited retinal dystrophy
GNB3	139130	Inherited retinal dystrophy
CYP4V2	608614	Inherited retinal dystrophy
ROM1	180721	Inherited retinal dystrophy
EFEMP1	601548	Inherited retinal dystrophy
ZNF408	616454	Inherited retinal dystrophy
RCBTB1	607867	Inherited retinal dystrophy
FZD4	604579	Inherited retinal dystrophy
LRP5	603506	Inherited retinal dystrophy
TSPAN12	613138	Inherited retinal dystrophy
OAT	613349	Inherited retinal dystrophy
CNNM4	607805	Inherited retinal dystrophy
CSPP1	611654	Inherited retinal dystrophy
TCTN2	613846	Inherited retinal dystrophy
B9D1	614144	Inherited retinal dystrophy
INPP5E	613037	Inherited retinal dystrophy
TTC21B	612014	Inherited retinal dystrophy
KIF7	611254	Inherited retinal dystrophy
TCTN1	609863	Inherited retinal dystrophy
TMEM237	614423	Inherited retinal dystrophy
CEP41	610523	Inherited retinal dystrophy
TMEM138	614459	Inherited retinal dystrophy
C5orf42	614571	Inherited retinal dystrophy
TCTN3	613847	Inherited retinal dystrophy
ZNF423	604557	Inherited retinal dystrophy
TMEM216	613277	Inherited retinal dystrophy
TMEM231	614949	Inherited retinal dystrophy
AHI1	608894	Inherited retinal dystrophy
ARL13B	608922	Inherited retinal dystrophy
CC2D2A	612013	Inherited retinal dystrophy

LRAT	604863	Inherited retinal dystrophy
C1QTNF5	608752	Inherited retinal dystrophy
CLUAP1	616787	Inherited retinal dystrophy
NPHP3	608002	Inherited retinal dystrophy
PEX1	602136	Inherited retinal dystrophy
GUCY2D	600179	Inherited retinal dystrophy
CEP290	610142	Inherited retinal dystrophy
RD3	180040	Inherited retinal dystrophy
RDH12	608830	Inherited retinal dystrophy
KCNJ13	603208	Inherited retinal dystrophy
SPATA7	609868	Inherited retinal dystrophy
AIPL1	604392	Inherited retinal dystrophy
LCA5	611408	Inherited retinal dystrophy
RPGRIP1	605446	Inherited retinal dystrophy
CRX	602225	Inherited retinal dystrophy
NMNAT1	608700	Inherited retinal dystrophy
DTHD1	616979	Inherited retinal dystrophy
ADIPOR1	607945	Inherited retinal dystrophy
MT-TL1	590050	Inherited retinal dystrophy
SOD2	147460	Inherited retinal dystrophy
BEST1	607854	Inherited retinal dystrophy
IMPG1	602870	Inherited retinal dystrophy
PRDM13	616741	Inherited retinal dystrophy
CDH3	114021	Inherited retinal dystrophy
IFT140	614620	Inherited retinal dystrophy
MAPKAPK3		Inherited retinal dystrophy
CAPN5	602537	Inherited retinal dystrophy
MFSD8	611124	Inherited retinal dystrophy
NDP	300658	Inherited retinal dystrophy
RP1L1	608581	Inherited retinal dystrophy
CTNNA1	116805	Inherited retinal dystrophy
MT-TH	590040	Inherited retinal dystrophy
TIMP3	188826	Inherited retinal dystrophy
ABCC6	177850	Inherited retinal dystrophy
RDH5	601617	Inherited retinal dystrophy
WDR19	608151	Inherited retinal dystrophy
KCNV2	607604	Inherited retinal dystrophy
CACNA2D4	608171	Inherited retinal dystrophy
CLCN7	602727	Inherited retinal dystrophy
HFE	613609	Inherited retinal dystrophy
ASRGL1	609212	Inherited retinal dystrophy
CEP164	614848	Inherited retinal dystrophy
GFAP	137780	Inherited retinal dystrophy
MIR204	610942	Inherited retinal dystrophy
RDH11	607849	Inherited retinal dystrophy
TUB	601197	Inherited retinal dystrophy
ITM2B	603904	Inherited retinal dystrophy
RBP4	180250	Inherited retinal dystrophy
LAMA1	150320	Inherited retinal dystrophy
PANK2	606157	Inherited retinal dystrophy
PRKCG	176980	Inherited retinal dystrophy
TRNT1	612907	Inherited retinal dystrophy
ABHD12	613599	Inherited retinal dystrophy
MT-TP	590075	Inherited retinal dystrophy

ANAPC1	608473	Inherited retinal dystrophy
EMC1	616846	Inherited retinal dystrophy
NEUROD1	601724	Inherited retinal dystrophy
OR2W3	616729	Inherited retinal dystrophy
SPP2	602637	Inherited retinal dystrophy
ADGRA3	612303	Inherited retinal dystrophy
AGBL5	615900	Inherited retinal dystrophy
ARL3	604695	Inherited retinal dystrophy
DHX38	605584	Inherited retinal dystrophy
EXOSC2	602238	Inherited retinal dystrophy
HK1	142600	Inherited retinal dystrophy
KIAA1549	613344	Inherited retinal dystrophy
KIZ	615757	Inherited retinal dystrophy
MVK	251170	Inherited retinal dystrophy
PRPF4	607795	Inherited retinal dystrophy
FLVCR1	609144	Inherited retinal dystrophy
RP1	603937	Inherited retinal dystrophy
IMPDH1	146690	Inherited retinal dystrophy
PRPF31	606419	Inherited retinal dystrophy
CRB1	604210	Inherited retinal dystrophy
PRPF8	607300	Inherited retinal dystrophy
TULP1	602280	Inherited retinal dystrophy
CA4	114760	Inherited retinal dystrophy
PRPF3	607301	Inherited retinal dystrophy
ABCA4	601691	Inherited retinal dystrophy
RP2	300757	Inherited retinal dystrophy
RPE65	180069	Inherited retinal dystrophy
OFD1	300170	Inherited retinal dystrophy
EYS	612424	Inherited retinal dystrophy
CERKL	608381	Inherited retinal dystrophy
NRL	162080	Inherited retinal dystrophy
FAM161A	613596	Inherited retinal dystrophy
RPGR	312610	Inherited retinal dystrophy
FSCN2	613596	Inherited retinal dystrophy
TOPORS	609507	Inherited retinal dystrophy
SNRNP200	601664	Inherited retinal dystrophy
SEMA4A	607292	Inherited retinal dystrophy
PRCD	610598	Inherited retinal dystrophy
NR2E3	604485	Inherited retinal dystrophy
MERTK	604705	Inherited retinal dystrophy
RHO	180380	Inherited retinal dystrophy
PDE6B	180072	Inherited retinal dystrophy
PROM1	604365	Inherited retinal dystrophy
KLHL7	611119	Inherited retinal dystrophy
PDE6A	180071	Inherited retinal dystrophy
RGR	600342	Inherited retinal dystrophy
CNGB1	600724	Inherited retinal dystrophy
IDH3B	604526	Inherited retinal dystrophy
SAG	181031	Inherited retinal dystrophy
GUCA1B	602275	Inherited retinal dystrophy
CNGA1	123825	Inherited retinal dystrophy
TTC8	608132	Inherited retinal dystrophy
C2orf71	613425	Inherited retinal dystrophy
ARL6	608845	Inherited retinal dystrophy

IMPG2	607056	Inherited retinal dystrophy
PDE6G	180073	Inherited retinal dystrophy
ZNF513	613598	Inherited retinal dystrophy
DHDDS	608172	Inherited retinal dystrophy
PRPF6	613979	Inherited retinal dystrophy
CLRN1	606397	Inherited retinal dystrophy
MAK	154235	Inherited retinal dystrophy
C8orf37	614477	Inherited retinal dystrophy
CDHR1	609502	Inherited retinal dystrophy
RBP3	180290	Inherited retinal dystrophy
NEK2	604043	Inherited retinal dystrophy
SLC7A14	615720	Inherited retinal dystrophy
PRPH2	179605	Inherited retinal dystrophy
IFT172	607386	Inherited retinal dystrophy
HGSNAT	610453	Inherited retinal dystrophy
RP9	607331	Inherited retinal dystrophy
GNPTG	607838	Inherited retinal dystrophy
ARL2BP	615407	Inherited retinal dystrophy
INVS	243305	Inherited retinal dystrophy
NPHP1	607100	Inherited retinal dystrophy
NPHP4	607215	Inherited retinal dystrophy
IQCB1	609237	Inherited retinal dystrophy
ELOVL4	605512	Inherited retinal dystrophy
TEAD1	189967	Inherited retinal dystrophy
OPN1SW	613522	Inherited retinal dystrophy
ADGRV1	602851	Inherited retinal dystrophy
WHRN	607928	Inherited retinal dystrophy
CEP250	609689	Inherited retinal dystrophy
MYO7A	276903	Inherited retinal dystrophy
USH1C	605242	Inherited retinal dystrophy
CDH23	605516	Inherited retinal dystrophy
PCDH15	605514	Inherited retinal dystrophy
USH1G	607696	Inherited retinal dystrophy
USH2A	608400	Inherited retinal dystrophy
PDZD7	612971	Inherited retinal dystrophy
HARS	142810	Inherited retinal dystrophy
CIB2	605564	Inherited retinal dystrophy
MT-TS2	590085	Inherited retinal dystrophy
VCAN	118661	Inherited retinal dystrophy
TLR2	603028	Inherited retinal dystrophy
RS1	300839	Inherited retinal dystrophy
PEX10	602859	Inherited retinal dystrophy
PEX11B	603867	Inherited retinal dystrophy
PEX12	601758	Inherited retinal dystrophy
PEX13	601789	Inherited retinal dystrophy
PEX14	601791	Inherited retinal dystrophy
PEX16	603360	Inherited retinal dystrophy
PEX26	608666	Inherited retinal dystrophy
PEX5	600414	Inherited retinal dystrophy
PEX6	601498	Inherited retinal dystrophy
PEX2	170993	Inherited retinal dystrophy
PEX7	601757	Inherited retinal dystrophy
SLITRK6	609681	Myopia
P3H2	610341	Myopia

LRPAP1	104225	Myopia
PRIMPOL	615421	Myopia
ZNF644	614159	Myopia
ACO2	100850	Optic neuropathies
MT-ND1	516000	Optic neuropathies
ATP1A3	182350	Optic neuropathies
RTN4IP1	610502	Optic neuropathies
UCHL1	191342	Optic neuropathies
AFG3L2	604581	Optic neuropathies
C12orf65	613541	Optic neuropathies
PRPS1	311850	Optic neuropathies
SLC25A46	610826	Optic neuropathies
OPA1	605290	Optic neuropathies
OPA3	606580	Optic neuropathies
TMEM126A	612988	Optic neuropathies
MFN2	608507	Optic neuropathies
SIX6	606326	Optic neuropathies
NBAS	608025	Optic neuropathies
PAX6	607108	Optic neuropathies
GP1BA	606672	Optic neuropathies
CISD2	611507	Optic neuropathies
NF2	607379	Optic neuropathies
NF1	162200	Optic neuropathies
COLEC11	612502	Others
MPZ	159440	Others
JAG1	601920	Others
NOTCH2	600275	Others
COL4A4	120131	Others
COL4A5	303630	Others
TP63	603273	Others
FGF10	602115	Others
SLC2A10	606145	Others
APTX	606350	Others
SETX	608465	Others
FOXC1	601090	Others
PITX2	601542	Others
ACTB	102630	Others
ACTG1	102560	Others
PTCH1	601309	Others
PTCH2	603673	Others
NSD1	606681	Others
NOD2	605956	Others
FOXL2	605597	Others
DRD5	126453	Others
PHF6	300414	Others
NR2F1	132890	Others
COL4A1	120130	Others
TFAP2A	107580	Others
ZNF469	612078	Others
PRDM5	614161	Others
CYLD	605018	Others
ABCA3	601615	Others
CCM2	607929	Others
ERCC2	126340	Others

ERCC1	126380	Others
CTC1	613129	Others
CHD7	608892	Others
SEMA3E	608166	Others
LYST	606897	Others
ERCC8	609412	Others
LONP1	605490	Others
PIGL	605947	Others
CTDP1	604927	Others
FBN2	612570	Others
POMT1	607423	Others
TMEM5	605862	Others
B3GALNT2	610194	Others
POMK	615247	Others
GMPPB	615320	Others
POMT2	607439	Others
POMGNT1	606822	Others
FKTN	607440	Others
FKRP	606596	Others
ISPD	614631	Others
POMGNT2	614828	Others
PLG	173350	Others
TIMM8A	300356	Others
EP300	602700	Others
FUCA1	612280	Others
ITGB3	173470	Others
CHN1	118423	Others
SALL4	607343	Others
ABCC2	601107	Others
ACD	609377	Others
NHP2	606470	Others
NOP10	606471	Others
TERT	187270	Others
WRAP53	612661	Others
EDARADD	606603	Others
PKP1	601975	Others
COL3A1	120180	Others
PLOD1	153454	Others
TNXB	600985	Others
LOXL1	153456	Others
MC1R	155555	Others
IKBKAP	603722	Others
FGFR1	136350	Others
FGFR2	176943	Others
FGFR3	134934	Others
LCAT	606967	Others
FRAS1	607830	Others
GRIP1	601993	Others
FREM2	608945	Others
FRMD7	300628	Others
GALK1	604313	Others
GALE	606953	Others
GALT	606999	Others
ANTXR1	606410	Others

GBA	606463	Others
ROBO3	608630	Others
GLB1	611458	Others
GM2A	613109	Others
HEXA	606869	Others
MLPH	606526	Others
RAB27A	603868	Others
JAM3	606871	Others
ACVRL1	601284	Others
ATL1	606439	Others
HPS1	604982	Others
AP3B1	603401	Others
HPS3	606118	Others
HPS4	606682	Others
HPS5	607521	Others
HPS6	607522	Others
DTNBP1	607145	Others
BLOC1S3	609762	Others
BLOC1S6	604310	Others
CBS	613381	Others
IDUA	252800	Others
FTL	134790	Others
GCM2	603716	Others
FAM126A	610531	Others
GJB2	121011	Others
IKBKG	300248	Others
RAX	601881	Others
GDF6	601147	Others
MFRP	606227	Others
PRSS56	613858	Others
GDF3	606522	Others
KIF21A	608283	Others
COL18A1	120328	Others
GALC	606890	Others
COX10	602125	Others
COX15	603646	Others
SURF1	185620	Others
OCRL	300535	Others
FREM1	608944	Others
MAP2K1	176872	Others
SIL1	608005	Others
TUBGCP4	609610	Others
TUBGCP6	610053	Others
VSX2	142993	Others
SHH	600725	Others
ABCB6	605452	Others
STRA6	610745	Others
ALDH1A3	600463	Others
DHODH	126064	Others
RYR1	180901	Others
GFER	600924	Others
ACTA2	102620	Others
TRIM37	605073	Others
B4GAT1	605517	Others

DAG1	128239	Others
LARGE1	603590	Others
AGRN	103320	Others
CHAT	118490	Others
COLQ	603033	Others
ADAMTS18	607512	Others
LAMB2	150325	Others
PPT1	600722	Others
CTSD	116840	Others
GRN	138945	Others
CTSF	603539	Others
TPP1	607998	Others
CLN3	607042	Others
DNAJC5	611203	Others
CLN6	608102	Others
CLN5	608102	Others
CLN8	607837	Others
MT-ATP6	516060	Others
ABHD5	275630	Others
NPC2	601015	Others
SMPD1	607608	Others
PTPN11	176876	Others
KRAS	190070	Others
SOS1	182530	Others
RAF1	164760	Others
NRAS	164790	Others
BRAF	164757	Others
IGBP1	300139	Others
HMX1	142992	Others
OCA2	611409	Others
TYRP1	115501	Others
SLC45A2	606574	Others
SLC24A5	609802	Others
C10orf11	614537	Others
GJA1	121014	Others
PABPN1	602279	Others
KCTD7	611725	Others
DMD	300377	Others
B3GLCT	610308	Others
PHOX2A	602753	Others
TUBA8	605742	Others
POLG	174763	Others
RNASEH1	604123	Others
SLC25A4	103220	Others
C10orf2	606075	Others
POLG2	604983	Others
RRM2B	604712	Others
DNA2	601810	Others
RGS9	604067	Others
RGS9BP	607814	Others
SLC4A4	603345	Others
PAX2	167409	Others
ZFYVE26	612012	Others
TREX1	606609	Others

PLK4	605031	Others
TINF2	604319	Others
KIAA0196	610657	Others
RECQL4	268400	Others
ESCO2	609353	Others
HESX1	601802	Others
ALDH3A2	609523	Others
MTPAP	613669	Others
SYNE1	608441	Others
ANO10	613726	Others
EEF2	130610	Others
ITPR1	147265	Others
KCNC3	176264	Others
KCND3	605411	Others
SPTBN2	604985	Others
SYT14	610949	Others
TGM6	613900	Others
TTBK2	611695	Others
WWOX	605131	Others
ATXN10	611150	Others
ATXN7	607640	Others
COL2A1	120140	Others
COL11A1	120280	Others
COL11A2	120290	Others
COL9A1	120210	Others
COL9A2	120260	Others
NAA10	300013	Others
VAX1	604294	Others
RARB	180220	Others
HMGB3	300193	Others
MAB21L2	604357	Others
BCOR	300485	Others
SOX2	184429	Others
OTX2	600037	Others
BMP4	112262	Others
HCCS	300056	Others
TTR	105210	Others
TCOF1	606847	Others
TUBB3	602661	Others
VHL	608537	Others
SMOC1	608488	Others
TYR	606933	Others
PAX3	606597	Others
MITF	156845	Others
SNAI2	602150	Others
EDNRB	131244	Others
EDN3	131242	Others
SOX10	602229	Others
RAB3GAP1	602536	Others
RAB3GAP2	609275	Others
RAB18	602207	Others
TBC1D20	611663	Others
WRN	604611	Others
ATP7B	606882	Others

NIPBL	608667	Others
HDAC8	300269	Others
GPR143	300808	Others
DNAJC19	608977	Others
SF3B4	605593	Others
PIK3R1	171833	Others
HGD	607474	Others
KCNJ10	602208	Others
MRE11A	600814	Others
ATM	607585	Others
ATP2C1	604384	Others
COL6A3	120250	Others
COL6A2	120240	Others
BLM	210900	Others
MTM1	300415	Others
CYP27A1	606530	Others
TFAP2B	601601	Others
DNM2	602378	Others
PDK3	300906	Others
ARSE	300180	Others
EBP	300205	Others
GNPAT	602744	Others
CNTN1	600016	Others
RAD21	606462	Others
SMC1A	300040	Others
ALDH18A1	138250	Others
ATP6V0A2	611716	Others
EFEMP2	604633	Others
LTBP4	604710	Others
PYCR1	179035	Others
PRX	605725	Others
DBH	609312	Others
ADAR	146920	Others
EMD	300384	Others
LMNA	150330	Others
SYNE2	608442	Others
LAMA3	600805	Others
MYH11	160745	Others
MYLK	600922	Others
PRKG1	176894	Others
ASAH1	613468	Others
PORCN	300651	Others
FMR1	309550	Others
CTSA	613111	Others
APC	611731	Others
KIF1BP	609367	Others
AGXT	604285	Others
GRHPR	604296	Others
HOGA1	613597	Others
PTH	168450	Others
STS	300747	Others
KDM6A	300128	Others
KMT2D	602113	Others
ATP13A2	610513	Others

SGCB	600900	Others
SGCD	601411	Others
TTN	188840	Others
COX7B	300885	Others
CREBBP	600140	Others
TGFBR1	190181	Others
TGFBR2	190182	Others
SMAD3	603109	Others
TGFB2	190220	Others
TGFB3	190230	Others
KCNH2	152427	Others
KCNJ2	600681	Others
TSC1	605284	Others
TSC2	191092	Others
MAN2B1	609458	Others
MANBA	609489	Others
GNAS	139320	Others
FLNA	300017	Others
AUH	600529	Others
MMACHC	609831	Others
ASPM	605481	Others
CDK5RAP2	608201	Others
CEP135	611423	Others
CEP152	613529	Others
ZNF335	610827	Others
TACO1	612958	Others
DYSF	603009	Others
MCOLN1	605248	Others
ARSB	611542	Others
GALNS	612222	Others
GNS	607664	Others
GUSB	611499	Others
IDS	300823	Others
NAGLU	609701	Others
MSH2	609309	Others
ITGA7	600536	Others
LAMA2	156225	Others
SEPN1	606210	Others
PLEC	601282	Others
CHRNG	100730	Others
DOK7	610285	Others
MSTN	601788	Others
SCN4A	603967	Others
DMPK	605377	Others
CNBP	116955	Others
LMX1B	602575	Others
NPC1	607107	Others
TPM2	190990	Others
TPM3	191030	Others
NEB	256030	Others
CFL2	601443	Others
SPINK5	605010	Others
PLA2G6	603604	Others
C19orf12	614297	Others

COASY	609855	Others
MID1	300552	Others
DDX59	615464	Others
TNFRSF11A	603499	Others
OSTM1	607649	Others
SNX10	614780	Others
TCIRG1	604592	Others
TNFSF11	602642	Others
SLC26A4	605646	Others
STK11	602216	Others
PGK1	311800	Others
KIT	164920	Others
CHMP1A	164010	Others
EXOSC3	606489	Others
RARS2	611524	Others
TSEN2	608753	Others
TSEN34	608754	Others
TSEN54	608755	Others
VRK1	602168	Others
UROD	613521	Others
PHYH	602026	Others
AGPS	603051	Others
TWIST1	601622	Others
ACTA1	102610	Others
FHL1	300163	Others
TRPV4	605427	Others
HSPG2	142461	Others
NEU1	608272	Others
DHCR7	602858	Others
NFIX	164005	Others
AP4M1	602296	Others
AP5Z1	613653	Others
B4GALNT1	601873	Others
CYP2U1	610670	Others
CYP7B1	603711	Others
DDHD2	615003	Others
ERLIN2	611605	Others
FA2H	611026	Others
GBA2	609471	Others
HSPD1	118190	Others
KIF1A	601255	Others
KIF5A	602821	Others
MARS2	609728	Others
NIPA1	608145	Others
REEP1	609139	Others
RTN2	603183	Others
SPG11	610844	Others
SPG7	602783	Others
ZFYVE27	610243	Others
MAPT	157140	Others
SALL1	602218	Others
POLR1C	610060	Others
BAP1	603089	Others
FAH	613871	Others

IRF6	607199	Others
TBX1	602054	Others
EPG5	615068	Others
ERCC3	133510	Others
XPA	611153	Others
XPC	613208	Others
RB1	614041	Retinoblastoma
