# Supplementary material for: Genotypic spectrum and phenotype correlations of EYS-associated disease in a Chinese cohort
Source: Eye (Lond). 2021 Oct 23;36(11):2122–9. doi: 10.1038/s41433-021-01794-6 (PMC9581949; doi:10.1038/s41433-021-01794-6)
Supplement: Supplementary file 5 — Supplementary Table 2. Genetic and demographic data of patients in this study [file 41433_2021_1794_MOESM5_ESM.pdf]

Supplementary Table 1. Genetic and demographic data of patients in this study

Patients ID	Gender	Age ranges	Nucleotide change	Amino acid change	Zygosis	Reported	Category	Diagnosis
F1-1	Female	26-30	c.4957_4958insA	p.Ser1653Lysfs2	Het	Yes	P	RP
			c.2528G>A	p.Gly843Glu	Het	No	LP	
F2-1	Male	36-40	c.8868C>A	p.Tyr2956*	Het	Yes	P	RP
			c.8608C>T	p.Arg2870*	Het	No	P	
F3-1	Male	26-30	c.7492G>C	p.Ala2498Pro	Het	Yes	P	RP
			c.4655T>A	p.Leu1552*	Het	No	P	
F4-1	Male	36-40	c.1750G>T	p.Glu584*	Hom	Yes	P	RP
F5-1			c.6416G>A	p.Cys2139Tyr	Het	Yes	P	RP
			c.4955C>A	p.Ser1652*	Het	Yes	P	
F6-1	Male	26-30	c.6416G>A	p.Cys2139Tyr	Het	Yes	P	RP
			c.8372T>C	p.Leu2791Pro	Het	No	LP	
F7-1	Male	36-40	c.629del	p.Tyr210Serfs47	Hom	No	LP	RP
			c.627A>T	p.Lys209Asn	Hom	No	LP	
F7-2	Female	36-40	c.629del	p.Tyr210Serfs47	Hom	No	LP	RP
			c.627A>T	p.Lys209Asn	Hom	No	LP	
F8-1	Male	31-35	c.8224C>T	p.Gln2742*	Het	No	P	RP
			c.7492G>C	p.Ala2498Pro	Het	Yes	P	
F8-2	Female	31-35	c.8224C>T	p.Gln2742*	Het	No	P	RP
			c.7492G>C	p.Ala2498Pro	Het	Yes	P	
F9-1	Male	51-55	c.7492G>C	p.Ala2498Pro	Het	Yes	P	RP
			c.6416G>A	p.Cys2139Tyr	Het	Yes	P	
F9-2	Female	56-60	c.7492G>C	p.Ala2498Pro	Het	Yes	P	RP
			c.6416G>A	p.Cys2139Tyr	Het	Yes	P	
F10-1	Male	66-70	c.8372T>C	p.Leu2791Pro	Het	No	LP	RP
			c.8170G>T	p.Glu2724*	Het	Yes	P	
F10-2	Male	66-70	c.8372T>C	p.Leu2791Pro	Het	No	LP	RP
			c.8170G>T	p.Glu2724*	Het	Yes	P	
F11-1	Female	36-40	c.8012T>A	p.Leu2671*	Het	Yes	P	RP
			c.7492G>C	p.Ala2498Pro	Het	Yes	P	
			c.6416G>A	p.Cys2139Tyr	Het	Yes	P	
F12-1	Male	21-25	c.6563T>C	p.Ile2188Thr	Het	No	VUS	RP
			c.1750G>T	p.Glu584*	Het	Yes	P	
F13-1	Male	16-20	c.6416G>A	p.Cys2139Tyr	Het	Yes	P	RP
			c.4912del	p.Arg1638GlufsX41	Het	No	LP	
			c.1649A>G	p.Tyr550Cys	Het	No	VUS	
F14-1	Male	46-50	c.6557G>A	p.Gly2186Glu	Het	Yes	P	RP
			c.5825G>T	p.Gly1942Val	Het	No	VUS	
F15-1	Male	31-35	c.7492G>C	p.Ala2498Pro	Het	Yes	P	RP
			c.4912del	p.Arg1638Glufs41	Het	No	LP	
F16-1	Female	46-50	c.7287C>A	p.Phe2429Leu	Het	No	VUS	RP
			c.2486del	p.Ile829Thrfs39	Het	No	LP	
F17-1	Male	26-30	c.8861T>C	p.Phe2954Ser	Het	No	LP	RP
			c.6544_6547del	p.Asn2182ValfsX2	Het	No	P	
F18-1	Female	51-55	c.8941_8956del	p.Met2981Leufs10	Het	No	LP	RP
			c.7882_7883del	p.Ser2628Trpfs3	Het	No	LP	
F18-2	Female	51-55	c.8941_8956del	p.Met2981Leufs10	Het	No	LP	RP
			c.7882_7883del	p.Ser2628Trpfs3	Het	No	LP	
F19-1	Male	41-45	c.6557G>A	p.Gly2186Glu	Het	Yes	P	RP
			EX23_31dup	CDS20_28	Het	Yes	P	
F19-2	Female	41-45	c.6557G>A	p.Gly2186Glu	Het	Yes	P	RP
			EX23_31dup	-	Het	Yes	P	
F20-1	Female	51-55	c.6557G>A	p.Gly2186Glu	Hom	Yes	P	RP
			c.2000G>A	p.Arg667His	Hom	Yes	LP	

F20-2	Male	51-55	c.6557G>A	p.Gly2186Glu	Hom	Yes	P	RP
			c.2000G>A	p.Arg667His	Hom	Yes	LP	
F21-1	Male	51-55	c.8608C>T	p.Arg2870*	Het	No	P	RP
			c.7492G>C	p.Ala2498Pro	Het	Yes	P	
F21-2	Male	46-50	c.8608C>T	p.Arg2870*	Het	No	P	RP
			c.7492G>C	p.Ala2498Pro	Het	Yes	P	
F22-1	Male	31-35	c.6416G>A	p.Cys2139Tyr	Hom	Yes	P	RP
F23-1	Male	31-35	c.4045C>T	p.Arg1349*	Het	Yes	P	RP
			EX20_26dup	CDS17_23dup	Het	Yes	P	
F24-1	Female	36-40	c.6416G>A	p.Cys2139Tyr	Het	Yes	P	RP
			c.2486del	p.Ile829Thrfs39	Het	No	LP	
F25-1	Male	41-45	c.8804_8805insA	p.Tyr2935*	Het	No	LP	RP
			EX20-22dup	-	Het	Yes	P	
F26-1	Male	66-70	c.4363A>G	p.Ile1455Val	Het	No	VUS	LCA
			c.4196T>C	p.Met1399Thr	Het	No	VUS	
F27-1	Male	31-35	c.6557G>A	p.Gly2186Glu	Het	Yes	P	RP
			c.6416G>A	p.Cys2139Tyr	Het	Yes	P	
F28-1	Male	31-35	c.8608C>T	p.Arg2870*	Het	No	P	RP
			c.476G>A	p.Cys159Tyr	Het	No	VUS	
F29-1	Male	41-45	c.7714G>T	p.Gly2572Cys	Het	No	LP	RP
			c.6557G>A	p.Gly2186Glu	Het	Yes	P	
F30-1	Female	26-30	c.9439_9442del	p.Leu3147Leufs3	Het	No	LP	RP
			c.564del	p.Lys188Asnfs69	Het	No	LP	
F31-1	Female	26-30	c.7492G>C	p.Ala2498Pro	Het	Yes	P	RP
			c.6416G>A	p.Cys2139Tyr	Het	Yes	P	
F32-1	Female	31-35	c.9439_9442del	p.Ile3148Asnfs2	Hom	No	LP	RP
			c.6976C>T	p.Arg2326*	Het	Yes	P	
F33-1	Female	46-50	c.6557G>A	p.Gly2186Glu	Het	Yes	P	RP
			c.4912del	p.Arg1638Glufs41	Het	No	LP	
F34-1	Male	11-15	c.8170G>T	p.Glu2724*	Het	Yes	P	RP
			c.6557G>A	p.Gly2186Glu	Het	Yes	P	
F35-1	Male	5-10	c.5474C>T	p.Thr1825Ile	Het	No	LP	LCA
			c.2953_2961del	p.*985_Gly987del	Het	Yes	P	
F36-1	Male	16-20	c.6557G>A	p.Gly2186Glu	Het	Yes	P	RP
			c.1182C>A	p.Cys394*	Het	No	P	
F37-1	Female	26-30	c.2892A>C	p.Glu964Asp	Het	Yes	VUS	RP
			c.2886C>G	p.Phe962Leu	Het	Yes	VUS	
F38-1	Female	26-30	c.8159T>C	p.Phe2720Ser	Het	No	LP	RP
			c.2486del	p.Ile829Thrfs39	Het	No	LP	
F39-1	Female	21-25	c.7630C>T	p.Leu2544Phe	Het	No	LP	RP
			c.5902G>A	p.Gly1968Arg	Het	No	LP	
F40-1	Female	36-40	c.6416G>A	p.Cys2139Tyr	Hom	Yes	P	RP
F41-1	Male	26-30	c.8012T>A	p.Leu2671*	Het	Yes	P	RP
			c.6416G>A	p.Cys2139Tyr	Het	Yes	P	
F42-1	Male	71-75	c.8923T>C	p.Phe2975Leu	Het	Yes	LP	RP
			c.2953_2961del	p.*985_Gly987del	Het	Yes	P	
F43-1	Male	31-35	c.7492G>C	p.Ala2498Pro	Het	Yes	P	RP
			c.8455del	p.Asp2819Ilefs2	Het	Yes	P	
F43-2	Female	56-60	c.8455del	p.Asp2819Ilefs2	Het	Yes	P	RP
			c.9026C>T	p.Thr3009Ile	Het	No	VUS	
F44-1	Female	41-45	c.9106_9107insTATA	p.Asn3036Ilefs2	Het	No	LP	RP
			c.2739-1G>A	_	Het	No	LP	
F45-1	Male	71-75	c.9311G>A	p.Gly3104Asp	Het	No	VUS	RP
			c.2486del	p.Ile829Thrfs39	Het	No	LP	

			c.307T>G	p.Leu103Val	Het	No	VUS	
F46-1	Male	41-45	c.5677_5681del	p.Tyr1893Argfs12	Het	No	LP	RP
			c.992C>T	p.Thr331Ile	Het	No	LP	

Het: heterozygous; Hom:homozygous; P:pathogenic; LP:likely pathogenic; VUS:variants of uncertain significance; RP:retinitis pigmentosa; LCA:Leber congenital amaurosis.
